# Integration of Artificial Intelligence in Biosensors for Enhanced Detection of Foodborne Pathogens

**DOI:** 10.3390/bios15100690

**Published:** 2025-10-12

**Authors:** Riza Jane S. Banicod, Nazia Tabassum, Du-Min Jo, Aqib Javaid, Young-Mog Kim, Fazlurrahman Khan

**Affiliations:** 1 Fisheries Postharvest Research and Development Division, National Fisheries Research and Development Institute, Quezon City 1128, Philippines; riza.banicod@nfrdi.da.gov.ph; 2 Marine Integrated Biomedical Technology Center, The National Key Research Institutes in Universities, Pukyong National University, Busan 48513, Republic of Korea; nazia99@pukyong.ac.kr (N.T.); 3 Research Center for Marine Integrated Bionics Technology, Pukyong National University, Busan 48513, Republic of Korea; 4 National Marine Biodiversity Institute of Korea (MABIK), Seochun 33662, Republic of Korea; dmjo@mabik.re.kr; 5 Interdisciplinary Program of Marine and Fisheries Sciences and Convergent Technology, Pukyong National University, Busan 48513, Republic of Korea; aqibj@pukyong.ac.kr; 6 Department of Food Science and Technology, Pukyong National University, Busan 48513, Republic of Korea; ymkim@pknu.ac.kr (Y.-M.K.); 7 Ocean and Fisheries Development International Cooperation Institute, Pukyong National University, Busan 48513, Republic of Korea; 8 International Graduate Program of Fisheries Science, Pukyong National University, Busan 48513, Republic of Korea

**Keywords:** artificial intelligence, foodborne pathogens, biosensor, food safety, food samples

## Abstract

Foodborne pathogens remain a significant public health concern, necessitating the development of rapid, sensitive, and reliable detection methods for various food matrices. Traditional biosensors, while effective in many contexts, often face limitations related to complex sample environments, signal interpretation, and on-site usability. The integration of artificial intelligence (AI) into biosensing platforms offers a transformative approach to address these challenges. This review critically examines recent advancements in AI-assisted biosensors for detecting foodborne pathogens in various food samples, including meat, dairy products, fresh produce, and ready-to-eat foods. Emphasis is placed on the application of machine learning and deep learning to improve biosensor accuracy, reduce detection time, and automate data interpretation. AI models have demonstrated capabilities in enhancing sensitivity, minimizing false results, and enabling real-time, on-site analysis through innovative interfaces. Additionally, the review highlights the types of biosensing mechanisms employed, such as electrochemical, optical, and piezoelectric, and how AI optimizes their performance. While these developments show promising outcomes, challenges remain in terms of data quality, algorithm transparency, and regulatory acceptance. The future integration of standardized datasets, explainable AI models, and robust validation protocols will be essential to fully harness the potential of AI-enhanced biosensors for next-generation food safety monitoring.

## 1. Introduction

Food safety is a pressing global problem that carries profound implications for public health, resource sustainability, and economic stability [1]. Foodborne illnesses, mainly due to bacterial pathogens such as *Salmonella*, *Campylobacter*, *Escherichia coli*, and *Listeria monocytogenes*, result in substantial morbidity and mortality worldwide [2,3]. The World Health Organization (WHO) estimated that, in 2010, foodborne diseases affected 600 million people, resulting in 420,000 deaths and 33 million disability-adjusted life years globally [4,5]. Diarrheal disease agents, particularly norovirus and *Campylobacter*, were the most frequent causes of illness, whereas non-typhoidal *Salmonella* accounted for the highest number of deaths [6].

Foodborne pathogens are present in diverse food matrices, including fresh produce, ready-to-eat products, seafood, meat, and poultry, where they may persist without visible signs of spoilage, stressing the need for rapid detection to ensure food safety and prevent outbreaks [7,8,9]. Conventional detection methods, predominantly culture-based techniques followed by biochemical identification, are inexpensive and simple, but they are time-consuming, often requiring two to three days for preliminary identification and more than a week for definitive species confirmation [10]. These problems have prompted the development of advanced, culture-independent techniques, including nucleic acid–based assays, immunological approaches, and biosensor-based systems capable of swift, and in some cases in situ, pathogen detection across various food samples [9,11].

Biosensor technologies deliver a faster, more portable, and potentially cost-effective alternative for food safety diagnostics by allowing timely pathogen identification directly at critical control points [12,13,14]. A biosensor is primarily composed of a biorecognition element, such as an antibody, enzyme, nucleic acid probe, or aptamer [15,16], immobilized onto a transducer that transforms the biological interaction into a measurable signal [10,17]. Depending on the transduction principle, biosensors can be categorized as optical, electrochemical, piezoelectric, or thermal, each having specific advantages when it comes to speed, portability, and suitability for on-site applications [9,18,19]. These systems can deliver results within minutes to hours, greatly reducing turnaround time compared to conventional culture-based methods [20]. Notwithstanding these advantages, several challenges hinder their broader adoption in routine food safety applications [21], which include interference from complex sample matrices [22], instability of bioreceptors under varying environmental conditions [23], non-specific binding resulting in false positives [24], difficulty in interpreting low-intensity or noisy signals [25], and the need for trained personnel to guarantee accurate operation and analysis [26].

**Figure 1:**
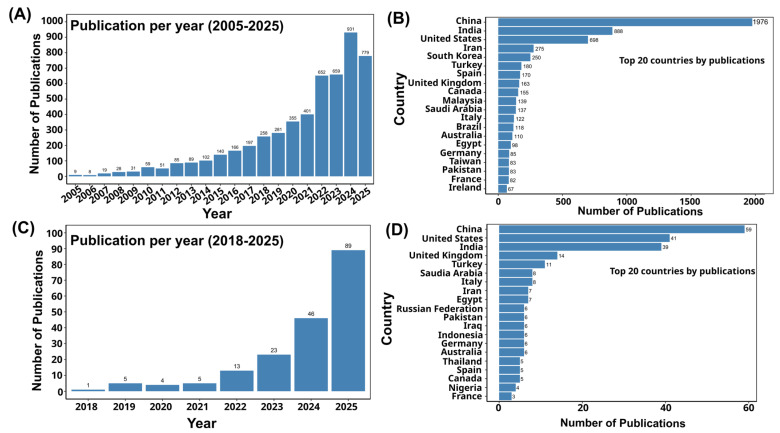
(**A**) The list of publications related to the biosensor-based detection of foodborne pathogens, which are indexed in Scopus, [Search within “all fields” and search documents using the keyword “biosensor-based detection of foodborne pathogens” from 2005 to 1 September 2025]. (**B**) Top 20 countries by publication related to the biosensor-based detection of foodborne pathogens. (**C**) The list of publications related to the artificial intelligence-based detection of foodborne pathogens, which are indexed in Scopus [Search within “all fields” and search documents using the keyword “artificial intelligence-based detection of foodborne pathogens” from 2018 to 1 September 2025]. (**D**) Top 20 countries by publications related to artificial intelligence-based detection of foodborne pathogens.

Artificial intelligence (AI) is transforming biosensing platforms for foodborne pathogen detection by means of advanced data processing, quantitative analysis, and real-time decision-making [27]. Machine learning models have been successfully applied to biosensor outputs for accurate pathogen classification and quantification in diverse food matrices, with reported accuracies exceeding 95% in some cases [28,29,30]. Artificial intelligence-driven approaches augment signal processing, suppress noise, and improve the sensitivity, selectivity, and stability of electrochemical, optical, and mass-based biosensors, thus supporting accurate detection even in complex food matrices [31,32]. Deep learning and convolutional neural networks (CNNs) have shown particular promise in applications such as surface-enhanced Raman spectroscopy (SERS)-based pathogen determination [33], microfluidic impedance flow cytometry for label-free bacterial classification [34], and digital microfluidic platforms for rapid, multiplex detection of viable pathogens [35]. These integrations allow biosensors to bypass laborious sample preparation, perform non-destructive spectroscopic analysis, and deliver accurate results in real time [36,37]. In addition, machine vision-assisted biosensors and AI-optimized sensor designs enhance portability and adaptability, making them well-suited for on-site and field applications [38]. These innovations enable accelerated and highly precise diagnostics, together with real-time monitoring, traceability, and predictive analytics, thus meeting the demands of a globalized food supply chain. The rapid growth of scientific literature on both biosensor-based and AI-enabled detection of foodborne pathogens reflects this accelerating interest, with publication trends and country-level contributions shown in Figure 1. This surge underscores the timeliness of a comprehensive review that consolidates advances in AI-integrated biosensing for food safety applications.

Artificial intelligence integration in biosensors aligns with current trends in digital health, smart agriculture, and Industry 4.0, where interconnected systems and data-driven insights guide decision-making [27,39]. Advances in microelectronics, wireless communication, cloud computing, and the Internet of Things (IoT) are driving the integration of intelligent food monitoring systems across supply chains, retail environments, and consumer settings. These smart biosensing platforms have the capacity to deliver real-time alerts, track contamination, and support predictive analytics for proactive food safety risk management [40,41].

In light of these developments, this review deals with the integration of AI into biosensor technologies for the enhanced detection of foodborne pathogens in diverse matrices. It discusses how AI is leveraged to improve biosensor capabilities, covering different detection platforms, biosensor types, operating principles, and strategies for enhancing analytical performance. Case studies from several food matrices are thoroughly examined to illustrate real-world applicability, alongside an evaluation of the remaining challenges and the future directions for advancing AI-enabled biosensing in food safety monitoring. As the global demand for safe, traceable, and high-quality food continues to surge, AI-assisted biosensors are well-placed to redefine food safety monitoring through unprecedented speed, accuracy, and connectivity.

## 2. Foodborne Pathogens and Challenges in Their Detection

Foodborne pathogens, including bacteria, viruses, and parasites, can contaminate food at several points along the supply chain, thus presenting a serious threat to public health by causing widespread illnesses and deaths [42,43]. Among them, *Norovirus*, *Campylobacter*, *Salmonella*, *L. monocytogenes*, and *E. coli* are particularly concerning owing to their high prevalence, severe clinical outcomes, and sustained involvement in foodborne outbreaks [2]. These pathogens not only colonize food products but can also release toxins and other harmful metabolites, some of which are resistant to inactivation during food processing [44]. Their persistence in food environments is aggravated by the ability to form biofilms on processing surfaces, which enhances resistance to cleaning, disinfection, and antimicrobial treatments [45,46]. Biofilms serve as protective barriers that shield pathogens from environmental stresses, thus complicating eradication efforts and increasing the risk of cross-contamination [47]. Notably, *L. monocytogenes* exhibits remarkable resilience by surviving under refrigeration, low pH, and high salt conditions, which allows it to thrive in food production and storage systems [48]. The risks extend across a wide variety of food matrices, such as fresh produce, seafood, meat, poultry, dairy products, and ready-to-eat meals, with the latter being particularly susceptible because of the absence of further cooking steps [7,49]. Accordingly, detection methods were developed to ensure compliance with statutory microbiological criteria, either zero-tolerance rules based on presence/absence or specified maximum permissible concentrations [50].

Reliable and highly sensitive detection of foodborne pathogens is critical to protecting public health [50], yet achieving this remains challenging due to the low abundance of pathogens, interference from background microbiota, and the complexity of food matrices [51]. A variety of detection techniques are currently employed, including traditional culture-based methods, immunological assays, nucleic acid-based approaches, and next-generation sequencing (NGS) [52,53,54].

Although culture-based methods remain the regulatory gold standard due to their low cost and ability to isolate live microorganisms, they are slow and often lack sensitivity, particularly for pathogens that are non-culturable or in a viable but non-culturable (VBNC) state [11,44,55]. Immunological assays like ELISA, lateral flow devices (LFDs), serotyping, and immunofluorescence are rapid methods that detect foodborne pathogens and their toxins by exploiting the specific binding affinity between microbial antigens and antibodies [56]. They are relatively easy to perform and highly specific, but their reliability can be compromised by matrix contamination, which may generate false positives [57].

Nucleic acid-based methods, such as polymerase chain reaction (PCR) and its variants, determine pathogens by amplifying specific DNA or RNA sequences using designed primers. They provide high sensitivity, reproducibility, and versatility, with the added advantage of quantifying and simultaneously detecting multiple targets; however, their application is limited by high costs, the need for specialized equipment, and susceptibility to inhibition from complex food components, which restrict routine use in low-resource settings [57,58]. Next-generation sequencing technologies are powerful tools in food safety because they provide detailed insights into pathogen genomes, virulence factors, and microbial community dynamics [59,60]. Despite their high resolution and broad detection capacity, their routine application in food testing laboratories is restricted by high costs, complex workflows, and the need for advanced bioinformatics expertise [61].

Additional complications arise as pathogens are unevenly distributed within food products [62], and natural food components such as fats, polysaccharides, and polyphenols can interfere with both nucleic acid and immunological assays. These matrix effects lower specificity, sensitivity, and reproducibility, thereby creating substantial barriers to accurate detection [63]. These problems have prompted the development of advanced, field-deployable diagnostic tools, with biosensors emerging as a particularly promising solution for quick, sensitive, and portable detection of foodborne pathogens across diverse food products [64]. As summarized in Table 1, each detection method presents unique trade-offs, with culture-based methods favored for regulatory compliance, molecular assays for sensitivity, and biosensors for portability. These comparisons highlight why AI integration is increasingly pursued to overcome limitations of conventional approaches.

## 3. Biosensor-Based Foodborne Pathogen Detection

Biosensors have emerged as advanced analytical platforms that overcome the drawbacks of conventional and molecular diagnostic methods by enabling rapid, sensitive, and portable detection of foodborne pathogens [70,79,80]. A biosensor is generally made up of a biorecognition element that selectively interacts with the target analyte, a transducer that converts this recognition event into a measurable physicochemical signal, and a signal processor that amplifies and interprets the output for qualitative or quantitative assessment [81]. In the context of food safety, biosensors make use of highly specific interactions between pathogens or their molecular markers and immobilized recognition elements, which allow real-time monitoring even in complex food matrices [82]. Their advantages include quick response times, usually within minutes to hours, on-site applicability, reduced dependence on centralized laboratories, as well as compatibility with point-of-care or miniaturized devices [83,84,85], thereby making them highly attractive for strengthening food safety surveillance.

According to International Union of Pure and Applied Chemistry (IUPAC) guidelines, biosensors can be classified according to their biological recognition mechanism or the physicochemical principle of signal transduction [86]. The biorecognition element is central to biosensor performance, as it determines specificity, sensitivity, and reproducibility [87]. High selectivity minimizes false positives and negatives, whereas strong affinity for target molecules enables the detection of even trace levels of pathogens [88]. In addition, their stability ensures a favorable signal-to-noise ratio in complex food environments [89], making them robust tools for food safety monitoring. Commonly used biorecognition elements include antibodies, aptamers, nucleic acids, enzymes, molecularly imprinted polymers (MIPs), bacteriophages, and cell receptors [90].

Antibodies are widely applied owing to their specificity, as shown in novel impedance immunosensors for the rapid and sensitive detection of *L. monocytogenes* in milk [91]. Nevertheless, their instability under temperature or pH variations [92] has led to the rise of aptamers or synthetic oligonucleotides generated via Systematic Evolution of Ligands by Exponential Enrichment (SELEX) that are stable, reusable, and chemically versatile. Conformational matching and intermolecular interactions are the key recognition mechanisms [93]. Aptamer-based microfluidic chips have facilitated the detection of *Bacillus cereus* at concentrations as low as 9.27 CFU/mL within one hour, highlighting their practicality in food testing [94]. Enzymes are biological macromolecules, primarily proteins, that act as highly efficient catalysts, thereby accelerating biochemical reactions by specifically recognizing and binding to their substrates [95]. A nanobody–horseradish peroxidase (Nb-HRP) sandwich ELISA developed by Gu et al. [96] applied this principle to achieve rapid and sensitive detection of *Salmonella enteritidis* in milk samples. In addition, MIPs, which are synthetic recognition materials that form template-shaped cavities within polymer matrices, can selectively capture pathogen surface markers through a lock-and-key mechanism. A particular example is the ratiometric electrochemical biosensor that integrates MIPs with aptamers, which showed ultrasensitive and self-calibrating detection of *Staphylococcus aureus* [97]. Bacteriophages or viruses that specifically infect bacteria have gained attention as biorecognition elements because their tail proteins bind to bacterial surface receptors with high strain-level specificity, allowing selective detection of viable pathogens [82]. Recent phage-based biosensors, including fluorescence and magnetic relaxation switching platforms, have demonstrated fast and sensitive detection of *Salmonella typhimurium* in food samples, achieving low detection limits while effectively avoiding interference from dead bacterial residues [38,98].

The transducer is equally important, as it interprets the biorecognition event into a quantifiable signal that determines the sensor’s analytical performance [99]. Electrochemical transducers, which operate via three primary measurement principles (potentiometric, amperometric, and impedimetric transduction mechanisms), are the most widely used in foodborne pathogen detection because of their high sensitivity, cost-effectiveness, and adaptability to portable systems [100,101]. An electrochemical aptamer-based biosensor quantified *Vibrio parahaemolyticus* in seafood at 5.74 CFU/mL in under 30 min [102]. Likewise, DNA-based electrochemical biosensors determined the presence of *Salmonella typhi* in milk and eggs with a detection limit of 1 CFU/mL [103], whereas a label-free electrochemical impedance spectroscopy (EIS) biosensor using a laser-induced graphene electrode functionalized with polyclonal antibodies was developed for the rapid detection of *S. enterica* Typhimurium in chicken broth within 22 min [104].

Optical transducers, which measure changes in fluorescence, color, surface plasmon resonance (SPR), or Raman scattering, are also powerful [105,106]. Advances in SERS-based biosensors made it possible to detect *S. aureus* at just 2 CFU/mL [107], while fiber-optic SPR systems identified *E. coli* O157:H7 in juice at 500 CFU/mL [108]. Other optical devices, such as quantum dot–based fluorescence biosensors, achieved multiplex detection of *Salmonella*, *S. aureus*, and *E. coli* in various food products [109,110,111]. Smartphone-integrated optical biosensors have also displayed portable, cost-effective detection of *E. coli* in milk and water, thereby accentuating their field applicability [112]. Piezoelectric transducers, even though less common, convert mass or acoustic changes into measurable signals [113]. Quartz crystal microbalance (QCM) biosensors have been successfully applied for *L. monocytogenes* determination in chicken meat and milk [114].

With substantial progress in nanomaterials, surface chemistry, and microfluidics [99,115], biosensors are rapidly evolving from experimental prototypes to practical tools for food safety assurance. Their ability to couple highly selective recognition with swift and sensitive transduction provides a pathway to reliable, field-deployable diagnostics [101]. When integrated with digital platforms and AI [30], biosensors are set to reshape food safety monitoring via real-time surveillance across global supply chains.

## 4. Artificial Intelligence in Biosensing Platforms

The increasing complexity of food matrices presents significant challenges for conventional biosensors, necessitating the adoption of advanced detection technologies. Food samples usually contain proteins, lipids, and carbohydrates that can compete with target analytes for binding sites on biosensor surfaces, thereby reducing assay specificity and sensitivity [116]. Moreover, biosensors frequently generate complex datasets characterized by signal variability, background noise, and matrix interferences, which complicate data interpretation and limit reliability in practical applications [117,118]. To overcome these problems, AI encompassing machine learning (ML) and deep learning (DL) delivers powerful solutions by allowing automated data analysis, pattern recognition, and predictive modeling [27,119]. In particular, ML algorithms can effectively process smaller and noisy datasets common in continuous monitoring applications, while simultaneously solving challenges such as electrode fouling, interference from non-target analytes, and sample inconsistencies [120].

Machine learning is a main branch of AI that allows computational systems to learn from data without being explicitly programmed [121]. It constitutes three main approaches, namely supervised, unsupervised, and reinforcement learning [122]. Supervised learning depends on labeled datasets, where input–output pairs guide model training with human oversight, which makes it relatively straightforward but largely reliant on curated training data [123]. Unsupervised learning, by contrast, detects hidden structures within unlabeled data, autonomously discovering patterns and groupings [124]. Meanwhile, reinforcement learning (RF) works via trial-and-error interactions with an environment to optimize decision-making to achieve defined objectives [125]. These ML approaches have been employed in biosensing for tasks such as pathogen classification, spectral data interpretation, as well as the development of semi-autonomous biosensing systems capable of adaptive responses in dynamic food environments [125,126].

Deep learning is an evolution of ML, which relies on multilayered artificial neural networks (ANNs) in order to learn hierarchical representations of data. As a high-dimensional reduction technique, DL is especially effective for complex function estimation in biosensing, functioning as a “black box” that transforms raw inputs into accurate predictions [127]. Unlike traditional ML approaches that depend heavily on manual feature extraction, DL models autonomously discover the relevant features for detection and classification through multiple layers of nonlinear transformations [128].

Artificial intelligence has the potential to influence each stage of biosensor development. It can aid in analyte selection by determining biomarkers with the highest discriminatory power, support the design of recognition elements through in silico modeling of binding interactions, improve signal transduction by optimizing sensor calibration, and streamline data analysis via rigorous classification and predictive algorithms [119].

### 4.1. AI-Assisted Selection and Multi-Analyte Analysis

Selecting appropriate analytes is essential to developing biosensors that can reliably detect foodborne pathogens, yet conventional approaches generally depend on trial-and-error experimentation that is time-intensive and may overlook subtle but critical biomarkers. Artificial intelligence has transformed this process by mining large-scale omics datasets, genomics, proteomics, and metabolomics, to determine candidate biomarkers with greater precision and efficiency. Machine learning algorithms can disclose hidden patterns, predict molecular interactions, and prioritize analytes with strong diagnostic potential while minimizing cross-reactivity [119,129]. For instance, lipopolysaccharides (LPSs) on the outer membrane of Gram-negative bacteria have been validated as biomarkers using a SERS platform incorporated with AI. Using principal component analysis and support vector machines, Abed et al. [130] successfully distinguished LPS “fingerprints” from *S. typhimurium* and some *E. coli* serotypes, thus achieving sensitive detection even within a complex food matrix like apple juice.

Artificial intelligence also allows multi-analyte profiling, where several indicators can be evaluated simultaneously to improve diagnostic sensitivity and specificity [119]. A recent example is a fluorescent sensor array based on carbon quantum dots (CQDs) functionalized with ampicillin, polymyxin, and gentamicin, each with distinct affinities for different bacterial targets. When the varied fluorescence responses were combined and analyzed via ML algorithms, the system achieved rapid, accurate, and highly sensitive identification of foodborne pathogens [131]. This underscores how AI-driven multi-analyte strategies can overcome the limitations of single-biomarker detection and improve biosensor performance in complex food environments.

### 4.2. Recognition Element Selection and Optimization

The choice of recognition elements is very important in biosensor design, as they determine the sensor’s capacity to selectively capture and detect foodborne pathogens. Recognition elements like antibodies, aptamers, enzymes, and synthetic polymers are traditionally discovered via experimental screening approaches such as SELEX, phage display, and high-throughput assays [132,133,134]. Systematic Evolution of Ligands by Exponential Enrichment has been the primary method for aptamer discovery since the 1990s, involving repeated cycles of screening and amplification [135,136]. However, conventional SELEX approaches are time-consuming, labor-intensive, and often fail to produce aptamers with performance comparable to antibodies [137].

To overcome these limitations, the optimization of recognition elements is shifting from traditional trial-and-error methods toward AI-guided molecular screening and structural modeling. In this approach, ML algorithms are used to forecast binding interactions, affinity levels, and stability under varying environmental conditions, providing a more streamlined pathway for biosensor development. Such strategies not only minimize the time and cost of research and development but also improve the reliability of detection in complex and heterogeneous food samples [138]. A stacked autoencoder-based deep neural network was used to analyze SERS spectra of *S. aureus*, enabling precise discrimination between methicillin-resistant and methicillin-sensitive strains with an accuracy of 97.99% and an AUC of 0.99 [139]. Likewise, Ding et al. [140] used a SERS-based biosensor with gold nanoparticles as the recognition surface, along with a multiscale CNN, to differentiate spectral fingerprints of *S. enteritidis*, *S. typhimurium*, and *S. paratyphi*, thus obtaining classification accuracies of above 97%. In addition, Ciloglu et al. [141] combined SERS probes with a k-nearest neighbors (k-NN) classifier to differentiate *S. aureus* and *Legionella pneumophila*, attaining 97.8% accuracy. These illustrate how AI-assisted recognition element optimization enhances biosensor specificity and robustness, allowing reliable detection of even closely related bacterial strains [142].

### 4.3. Transducer Design

At the transduction stage, AI enhances biosensor performance through the intelligent design of advanced materials, the miniaturization of instrumentation, and the development of novel sensing strategies that move beyond traditional biomolecular interactions [143]. Since electrode and optical materials directly influence sensitivity and selectivity, material design is fundamental to biosensor function [119]. Artificial intelligence algorithms excel at analyzing huge datasets to predict material properties and support the de novo design of next-generation biomaterials with optimized transduction characteristics [144,145].

Artificial intelligence also contributes to refining the functional behavior of transducers. A “Bidirectional” bacterial imprinting photoelectrochemical (BIPs-PEC) biosensor has been developed for the highly selective detection of *S. aureus* in complex food matrices. This platform combined bacterial imprinting with a dual-mode PEC system, where positive and negative bias voltages produced complementary signal outputs. Molecular docking and ML models were used to predict binding interactions and optimize electron transfer, which reduces matrix interference and improves analytical precision [146].

Instrument miniaturization is another area where AI has become important, as conventional components such as spectrometers or potentiostats are typically bulky and unsuitable for field deployment. Through advanced computational methods, AI allows compact devices to achieve accuracy comparable to laboratory-grade instruments. A multiplex system that integrates a low-cost three-dimensional nanostructure swab with a portable Raman spectrometer and CNN analysis was developed by Kang et al. [147]. The platform rapidly determined *E. coli* O157:H7, *S. enteritidis*, *L. monocytogenes*, *S. aureus,* and *Yersinia enterocolitica* within five minutes, even on contaminated food surfaces and utensils, achieving performance equivalent to conventional laboratory methods.

Artificial intelligence has also been applied to the design of biorecognition-free transducers, which operate even without specific molecular recognition elements like antibodies or aptamers. A nanosensor array constructed from aggregation-induced emissive nanosilicons exemplifies this approach, where ML algorithms, including ANNs, facilitated the discrimination of eight foodborne pathogens with 93.75 to 100% accuracy within one hour [148].

### 4.4. Signal Processing and Interpretation

Effective signal processing and interpretation are vital for translating raw biosensor outputs into meaningful diagnostic information [149]. In food safety monitoring, biosensors sometimes produce complex signals influenced by noise, background interference, and variability across food matrices [116]. Traditional signal handling approaches, such as baseline correction or filtering, are generally inadequate for reliably distinguishing target signals from irrelevant fluctuations. Artificial intelligence provides a powerful alternative by enabling advanced methods to clean, process, and interpret biosensor signals with high precision [150].

At the data acquisition stage, AI can improve sensor performance in real time. Reinforcement learning, for instance, has been utilized to dynamically adjust operational parameters, such as light intensity or exposure time in optical sensors, to prevent saturation, improve signal quality, and extend biosensor usability across changing environmental conditions. These feedback-driven systems enable continuous calibration without physical redesign of the sensor, which makes them particularly valuable for point-of-care and in-field applications [151,152].

During signal processing, AI plays a critical role in managing the vast, high-dimensional datasets typical of biosensing platforms. Machine learning algorithms can make use of dimensionality reduction techniques to simplify data without compromising diagnostic accuracy, such as converting complex electrochemical impedance spectra into concise, interpretable formats [153]. Adaptive filtering methods powered by neural networks enhance biosensor reliability by distinguishing true signals from noise with high accuracy, ensuring that relevant pathogen-specific information is not lost during preprocessing. Artificial intelligence-driven adaptive sampling has also been employed to determine optimal data collection times, thus minimizing unnecessary measurements while maximizing the extraction of informative signals [154].

In the analysis phase, AI models, such as support vector machines, decision trees, random forests, and neural networks, are being used to classify and quantify pathogens on the basis of biosensor outputs [155,156,157]. These models can discern subtle differences in impedance patterns, spectral signatures, or fluorescence intensities that traditional methods may overlook. Deep learning frameworks, especially CNNs, are largely effective for analyzing spectroscopic data, such as Raman or fluorescence spectra, by automatically extracting features and enabling rapid pathogen discrimination [150]. Likewise, recurrent neural networks (RNNs) and related models have been used for time-series analysis in continuous biosensing systems, thus supporting real-time pathogen monitoring in food production environments [158].

Lastly, at the interpretation stage, AI allows the incorporation of biosensor outputs into broader diagnostic frameworks. Algorithms can point out anomalous patterns and even predict future contamination trends based on historical data [159]. More advanced applications combine biosensor data with other heterogeneous sources, such as genomic data, supply chain monitoring, as well as environmental conditions, to provide a more holistic assessment of food safety risks.

## 5. Integration of AI with Biosensors for Enhanced Detection of Foodborne Pathogens

The integration of AI into biosensor platforms is driving a new generation of food safety diagnostics [150]. While biosensors already provide rapid and sensitive detection of foodborne pathogens, their performance can be compromised by signal noise, variability in sensitivity, and interference from complex food matrices [116]. Artificial intelligence mitigates these challenges by enabling advanced signal processing, robust pattern recognition, and predictive modeling, thereby enhancing biosensor reliability and applicability under real-world conditions [27].

**Figure 2:**
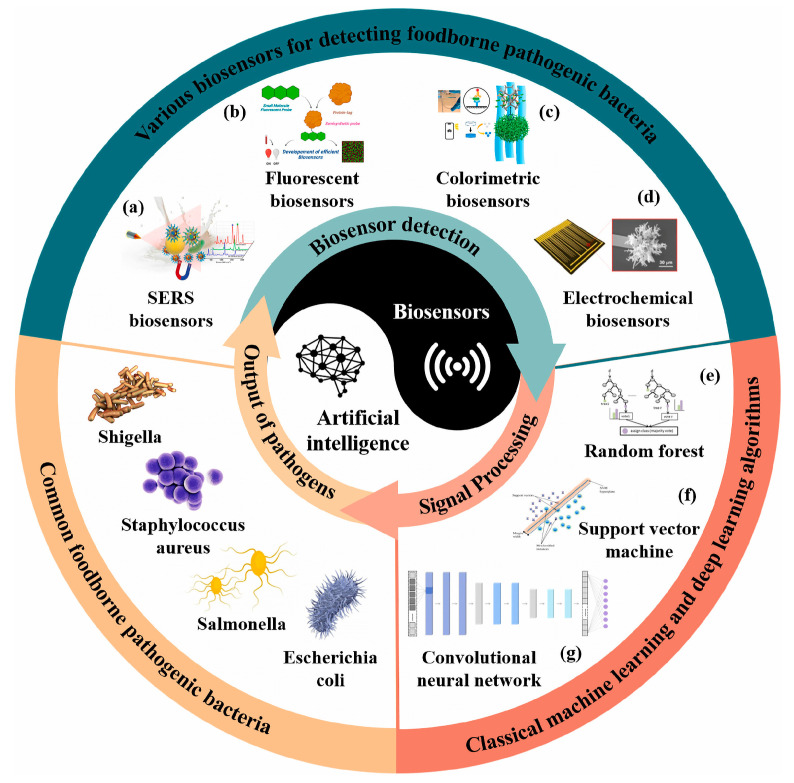
Conceptual framework illustrating the integration of artificial intelligence into biosensor platforms for foodborne pathogen detection. (**a**–**d**) Various biosensors for detecting foodborne pathogens: (**a**) SERS biosensors, (**b**) Fluorescent biosensors, (**c**) Colorimetric biosensors, and (**d**) Electrochemical biosensors. (**e**–**g**) Classical machine learning and deep learning algorithms for signal processing and pathogen classification: (**e**) Random forest, (**f**) Support vector machine, and (**g**) Convolutional neural network. Reprinted with permission from [150]. Copyright © 2025 Elsevier Ltd. A.

The choice of AI algorithm is closely linked to the biosensor’s data modality and intended task. Since biosensors operate on different transduction principles and generate diverse outputs, the strategies for incorporating AI may vary substantially. Each algorithm excels in particular scenarios, enhancing accuracy, robustness, and quantification depending on the type of signal analyzed [150]. Figure 2 provides an overview of this integration, highlighting common foodborne pathogens, biosensor platforms, and the AI algorithms most frequently employed.

Machine learning algorithms apply statistical principles to identify patterns for classification, regression, and clustering, and are most effective with smaller, structured datasets. Common approaches include linear and logistic regression for predictive modeling, decision trees (DT) for hierarchical classification, random forest (RF) that integrates multiple DTs to reduce overfitting, support vector machines (SVMs) for margin-based classification, K-nearest neighbors (KNN) for instance-based learning, Naïve Bayes (NB) for probabilistic classification, K-means for clustering, and principal component analysis (PCA) for dimensionality reduction [150,160]. Among supervised methods, SVM enhances discrimination of complex signals and supports real-time impedimetric sensing on mobile platforms [161], while RF improves robustness and accuracy in spectroscopic, electrochemical, and imaging-based detection [162]. Decision trees provide interpretable, rule-based classification [29] while KNN achieves effective pattern recognition for refractive index and microbial detection [163]. Linear discriminant analysis (LDA) improves separability in overlapping datasets, enhancing classification performance [164]. Unsupervised learning methods such as PCA and hierarchical clustering analysis (HCA) are valuable for high-dimensional biosensor data. Principal component analysis projects correlated variables into orthogonal components, simplifying interpretation and improving classification accuracy [165], while HCA organizes data into nested clusters, enhancing selectivity and enabling target discrimination in multi-array systems [166].

Regression-based models support quantitative biosensing, with partial least squares regression (PLSR) handling high-dimensional, correlated predictors to improve detection precision and reduce error [167] and Bayesian ridge regression (BRR) applying Bayesian inference to prevent overfitting and quantify uncertainty, achieving high predictive accuracy in optical biosensors [168]. XGBoost, a gradient boosting ensemble method, offers superior predictive power and interpretability, which performs well in complex and imbalanced datasets [169].

Deep learning methods extend biosensor capabilities by automatically extracting hierarchical features from raw data, making them particularly effective for large-scale and high-dimensional datasets. Artificial neural networks (ANNs/MLPs) capture nonlinear relationships in mixed bacterial samples, CNNs extract spatial features from fluorescence and Raman images to achieve low detection limits, and RNN models temporal dependencies for real-time monitoring. Advanced object-detection models such as YOLO and Faster R-CNN identify and quantify weak signals in colorimetric or fluorescence assays, thereby significantly enhancing sensitivity and accuracy. Despite their “black-box” nature, DL models offer strong generalization and adaptability, thus improving robustness and reliability across diverse biosensor platforms [150,170]. Table 2 presents representative studies where AI integration has consistently improved accuracy, lowered detection limits, enhanced linearity, and reduced detection time.

### 5.1. Electrochemical Biosensors

Electrochemical biosensors are among the most widely investigated platforms for the detection of foodborne pathogens owing to their high sensitivity, portability, and likelihood for miniaturization [118,150]. Their operation relies on the conversion of a biological recognition event into an electrical signal. A biorecognition element, such as an antibody, enzyme, nucleic acid probe, aptamer, or bacteriophage, is immobilized onto an electrode surface. When a pathogen binds to this recognition layer, the electrochemical properties of the electrode interface change, producing measurable signals, which are classified as current, potential, impedance, or conductivity. These signals correspond directly with the presence and concentration of the target organism. Among these, EIS has received particular attention owing to its label-free operation and suitability for real-time monitoring in complex food matrices [101]. Notwithstanding their promise, several limitations hinder routine adoption, including the requirement for skilled operation, potential loss of enzyme activity in enzymatic sensors, variability in immobilization processes, and signal interference from heterogeneous food samples [118,182]. In addition, many systems confront challenges in reproducibility, validation with large-scale food samples, and scalability for multiplexed detection [183].

The integration of AI into electrochemical biosensors offers powerful solutions to these barriers. Traditional ML algorithms, such as SVM and RF, have proven effective for feature extraction and classification when working with smaller datasets, thus allowing for accurate determination of pathogens with modest computational demands. The B.EL.D™ device, based on a bioelectric recognition assay, incorporates electrochemical signal readouts with AI-driven classification algorithms to determine *Salmonella* spp. in meat. It acquired accuracies of up to 97.7% with a detection limit of 0.6 log CFU/g, and delivered results substantially faster than standard ISO methods because it requires only a 24 h enrichment step followed by a rapid 3 min analysis [176]. Similarly, cell-imprinted EIS sensors have deployed RF models to discriminate and semi-quantify *E. coli*, *S. aureus*, and *V. parahaemolyticus* across a concentration range of 10^1^–10^6^ CFU/mL, demonstrating promising performance in both laboratory-prepared and real samples [177]. An electronic tongue based on EIS has also been applied for the detection of *S. aureus* in milk and for the diagnosis of bovine mastitis. The system employed layer-by-layer films composed of chitosan, chondroitin sulfate, sericin, and gold nanoparticle/sericin composites, which have shown differential molecular-level interactions with the target pathogen. Capacitance data were analyzed using ML algorithms, particularly DT models, to construct multidimensional calibration spaces capable of discriminating between pathogen concentrations and potential interferents. The sensor array obtained limits of detection from 2.01 to 3.41 CFU/mL in blank milk depending on the sensing unit, and when combined with ML-based classification, it delivered 100% accuracy in distinguishing *S. aureus* concentrations under controlled conditions [178].

In contrast, DL approaches are increasingly employed when electrochemical biosensors generate large and complex datasets. Aliev et al. [184] created an electrochemical platform using gallium–indium alloys and hydrogels, with multilayer perceptron networks applied to cyclic voltammetry data. The model achieved 97% accuracy, a precision–recall of 0.992, and a ROC AUC of 1.00 in measuring *E. coli* across concentrations from 10^2^ to 10^9^ CFU/mL. While gradient boosting and RF performed slightly better after preprocessing, the scalability and flexibility of DL models underline their adaptability to diverse biosensing contexts and their potential in multi-pathogen detection. Although ML is a strong choice for smaller datasets and faster analysis, DL presents distinct advantages in handling high-dimensional data, subtle signal variations, and complex sample matrices [150].

### 5.2. Optical Biosensors

Optical biosensors have emerged as powerful analytical tools for foodborne pathogen detection owing to their ability to deliver quick, label-free, and highly sensitive monitoring of biomolecular interactions. Their fundamental principle is based on transducing the interaction between a target pathogen and a biorecognition element into an optical signal that can be correlated with the presence and concentration of the pathogen. Upon binding, these interactions incite changes in optical properties like absorption, reflection, refraction, fluorescence, chemiluminescence, or Raman scattering, which are subsequently measured by the transducer. Compared to conventional culture or immunological methods, optical biosensors have faster turnaround, lower detection limits, and the potential for integration into point-of-care systems, thus making them highly attractive for food processing applications [80,105].

However, fabrication of sophisticated optical devices often involves high costs and requires advanced instrumentation, which may not be practical in resource-limited settings. In addition, interference from complex food matrices, instability of immobilized biorecognition elements, as well as reproducibility issues present substantial barriers to real-world application [185]. Addressing these problems through integration with nanomaterials and AI for data processing holds promise for advancing optical biosensors from proof-of-concept to scalable food safety solutions.

#### 5.2.1. Colorimetric Biosensor

Colorimetric biosensors depend on visible changes caused by nanoparticle aggregation or enzymatic reactions, often enabling detection by the naked eye without sophisticated instrumentation. To improve digital analysis, standardized models like RGB and HSV have been used to ensure consistent color representation across display systems [186]. Despite that, these traditional approaches are sensitive to lighting, background interference, and image capture conditions, and often require manual adjustments that are inefficient and subject to human error. Artificial intelligence addresses these challenges by automating the processing and interpretation of colorimetric signals to lessen subjectivity and enhance precision in complex, multivariate sample environments [150]. Pattern recognition and advanced data analytics enable AI-driven colorimetric biosensors to obtain greater accuracy [187], while integration with portable platforms such as smartphones further strengthens real-time applicability, as mobile devices provide both powerful computational resources and convenient imaging capabilities [188].

Yang et al. [78] developed a paper-based chromogenic array impregnated with 23 dyes to capture volatile organic compounds (VOCs) transmitted by viable foodborne pathogens. Using a multilayer neural network, the resulting complex colorimetric patterns were analyzed with accuracies of 91–95% for distinguishing *E. coli*, *L. monocytogenes*, and *S. aureus* in fresh-cut lettuce that was subjected to temperature abuse. A deep feed-forward neural network coupled with a paper-based colorimetric array also allowed the detection of pathogen-specific VOCs in shredded cheddar cheese, achieving 85–92% accuracy at 3–5 log CFU/g and 72–96% at 1 log CFU/g within 24 h, without requiring enrichment or incubation [173]. Likewise, in ground chicken, chromogenic dye interactions with VOCs were analyzed to detect *L. monocytogenes*, *S. enterica*, and *E. coli* O157:H7, with the system having more than 90% accuracy at contamination levels as low as 1 log CFU/g within 5–7 h at 25 °C, and maintaining greater than 80% accuracy within 24 h at 4 °C [174].

**Figure 3:**
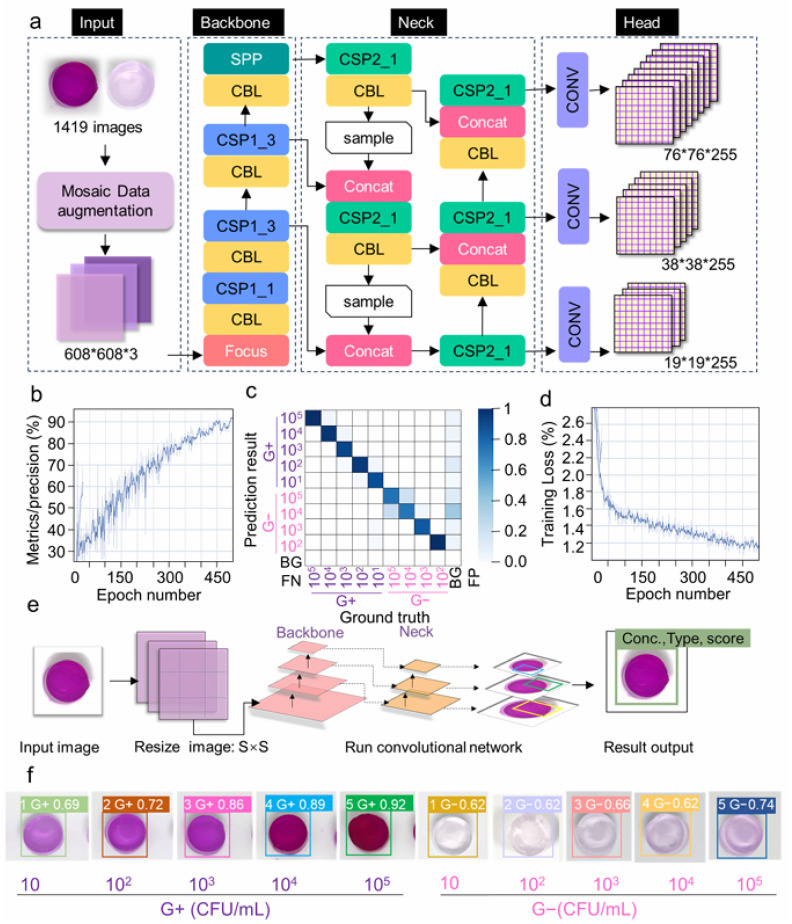
Integration of YOLOv5 for AI-assisted biosensor analysis: (**a**) Structure of the YOLOv5 network, including input, backbone, neck, and head modules for feature extraction and classification. (**b**) Precision curve showing model performance during training. (**c**) Confusion matrix illustrating classification accuracy across categories. (**d**) Training loss curve indicating model optimization. (**e**) Workflow of the YOLOv5 algorithm, from image acquisition and resizing to convolutional processing and final output. (**f**) Example detection results showing Gram-positive and Gram-negative bacteria with their concentrations and confidence scores [175]. Copyright © 2024 Elsevier B.V.

Cui et al. [175] advanced the field by creating an AI-assisted smartphone-based colorimetric biosensor that targets hyaluronidase, which are enzymes secreted during bacterial infection. The system employed a hydrogel-based bioreactor and signal generator, where enzyme-mediated degradation brought color changes analyzed by a YOLOv5 algorithm on a smartphone interface (Figure 3). This platform has an ultra-low detection limit of 10 CFU/mL within 60 min and successfully distinguishes between Gram-positive and Gram-negative bacteria, as well as live and dead cells. Its performance was validated in blueberries, antimicrobial susceptibility testing, as well as clinical specimens, recording sensitivity and specificity values of 100%.

#### 5.2.2. Fluorescent Biosensors

Fluorescent biosensors are analytical devices that detect microorganisms and biomolecules by measuring light emission from fluorescent labels such as dyes, proteins, or quantum dots when excited by light [189]. These systems provide quantitative or qualitative information contingent upon changes in signal intensity, wavelength, or lifetime, thus making them popular for foodborne pathogen detection because of their speed, sensitivity, and ease of visualization [150]. A major drawback of conventional fluorescent biosensors is their “lock-and-key” design, which restricts them to recognizing only one pathogen at a time, whereas background interference in complex food samples further reduces signal quality [190]. Artificial intelligence offers a solution by automating image and signal analysis, distinguishing true signals from noise, and applying pattern recognition to classify multiple pathogens simultaneously, thereby greatly enhancing the accuracy, efficiency, and practicality of fluorescent biosensors [150].

**Figure 4:**
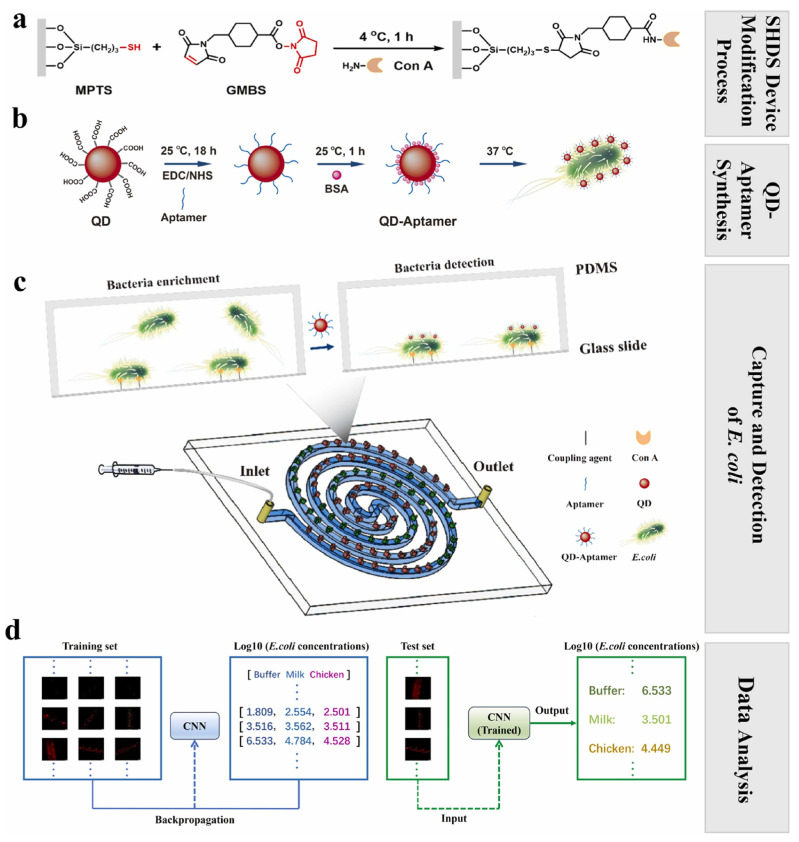
Workflow of the SHDS microfluidic biosensor integrated with AI for rapid detection of *Escherichia coli*: (**a**) Chemical modification of the microfluidic device surface with Con A. (**b**) Preparation of QD–aptamer fluorescent probes for selective binding to *E. coli.* (**c**) Operation of the spiral SHDS microfluidic biosensor, showing bacterial enrichment and detection steps within the device. (**d**) Application of a convolutional neural network (CNN) for training, validation, and prediction of *E. coli* concentrations from processed fluorescence images in buffer, milk, and chicken samples. Reprinted from [155]. Copyright © 2025 Elsevier B.V.

Ma et al. [155] designed a DL-assisted staggered herringbone double-spiral (SHDS) microfluidic biosensor that coupled lectin-based bacterial capture with quantum dot–aptamer probes and a ResNet-18 CNN for image analysis (Figure 4). This platform has an impressive detection limit of 2 CFU/mL within 1.5 h, with more than 99% predictive accuracy in both milk and chicken samples, as it effectively overcomes background interference and delivers reliable quantification in food matrices. Similarly, Zhang et al. [156] created a perovskite quantum dot (PQD)-based fluorescent sensor array analyzed by SVM algorithms that allow for accurate classification of *E. coli*, *S. aureus*, *S. typhimurium*, *L. monocytogenes*, and *P. aeruginosa* within minutes across 10^3^–10^7^ CFU/mL. Remarkably, this system not only detected but also sterilized pathogens, inactivating more than 99% of bacteria post-detection, highlighting its multifunctional potential for food safety monitoring. Xiao et al. [131] reported a CQD-based fluorescent sensor array functionalized with antibiotics that prompted unique quenching patterns for different pathogens. The platform achieved 100% classification accuracy by applying ML algorithms, including KNN, Naive Bayes, DT, and SVM in both pure cultures and spiked food matrices, and successfully identified mixed bacterial populations at concentrations as low as 10^3^ CFU/mL.

#### 5.2.3. Surface Plasmon Resonance

Surface plasmon resonance (SPR) biosensors are optical devices that utilize surface plasmon–polaritons to monitor interactions between analytes in solution and biomolecular recognition elements immobilized on a sensor surface [191]. These label-free, real-time analytical systems detect refractive index changes related to binding events, thus allowing sensitive monitoring of pathogen–receptor interactions [192]. Surface plasmon resonance platforms have been successfully applied to determine major foodborne pathogens, including *E. coli* O157:H7, *Salmonella* spp., *L. monocytogenes*, and *Campylobacter jejuni* across diverse food matrices [193,194,195].

However, detection limits often range between 10^3^ and 10^7^ CFU/mL, which necessitates enrichment steps to achieve clinically relevant sensitivity [196]. Complex food matrices usually cause surface fouling, although recent progress in ultra-low fouling coatings, such as poly (carboxybetaine acrylamide) brushes, has shown promise in mitigating this issue [193]. Multiplex detection is still difficult due to cross-reactivity and interference among coexisting bacterial species [195]. In addition, variations in antibody immobilization efficiency and matrix-dependent factors like pH can substantially affect specificity and reproducibility [194].

Artificial intelligence offers powerful strategies to deal with these limitations, with ML algorithms streamlining signal interpretation, shortening analysis times, and improving overall diagnostic accuracy [197]. In particular, DNNs coupled with spectral subtraction techniques have been shown to largely enhance signal-to-noise ratios, enabling more accurate detection of low-concentration analytes in portable wavelength-based SPR systems [198].

#### 5.2.4. Surface-Enhanced Raman Scattering (SERS)

Surface-enhanced Raman scattering biosensors are optical detection systems that amplify weak Raman signals by placing target molecules onto metal nanostructures with strong electromagnetic fields, enabling highly sensitive analysis at trace levels. Their advantages, such as low cost, rapid detection, minimal fluorescence interference, and compatibility with aqueous environments, make them a promising platform for foodborne pathogen monitoring. Nevertheless, practical applications are constrained by limited selectivity in complex food matrices, where overlapping spectral features from multiple components or closely related bacterial species complicate accurate identification [199].

To address these issues, label-based strategies, which utilize SERS-active tags for direct quantification, and label-free approaches, which rely on intrinsic bacterial spectral fingerprints but demand advanced computational support, have been employed. In both cases, AI techniques play a critical role by reducing data dimensionality, removing noise, and extracting distinctive spectral features, thereby improving specificity and enabling reliable pathogen classification even in challenging sample environments [200].

**Figure 5:**
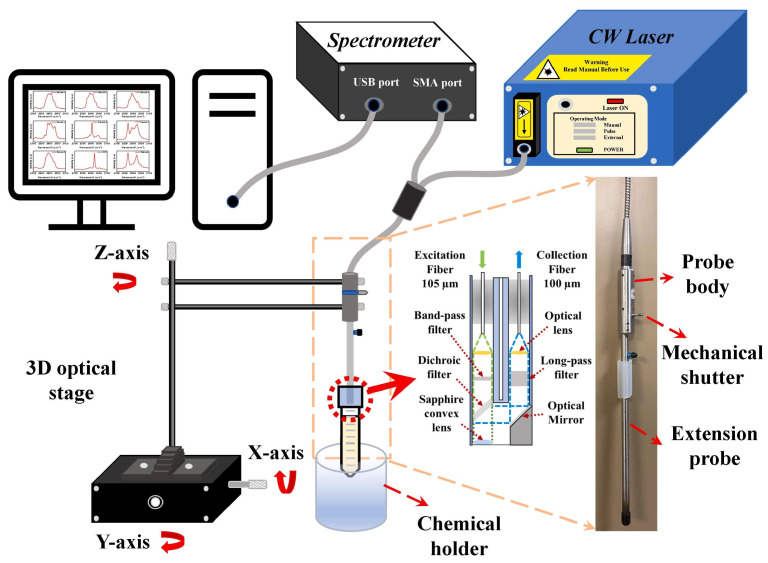
Schematic representation of the fiber-optic Raman spectroscopy system. The setup consists of a continuous-wave laser source connected to a Raman spectrometer and a fiber-optic probe mounted on a three-axis optical stage above a chemical holder. The probe is coupled to the excitation source and spectrometer through separate optical fibers, enabling remote measurement of samples. The inset in the middle illustrates the internal optical configuration of the probe, including filters and lenses for excitation and signal collection, while the image on the right highlights the physical structure of the probe body with its extension and mechanical shutter. The Raman spectra collected by this system were subsequently processed using AI models to extract discriminative features and achieve accurate classification of pathogen-related volatile compounds. Reprinted with permission from [179]. Copyright © 2023 Elsevier Ltd.

Zhang et al. [179] developed a portable fiber-optic Raman probe (Figure 5) for real-time detection of VOCs associated with *E. coli*, *L. monocytogenes*, and *Salmonella* spp. The system enabled in situ spectral acquisition from complex mixtures, even at high dilutions (up to 100-fold). To translate these complex spectra into accurate pathogen classification, they integrated six supervised ML models, including RF, SVM, and ANN, which processed the spectral fingerprints to extract key features and discriminate between bacterial groups. The ANN achieved 95% test accuracy with an AUC of 0.99 for methanol-diluted samples, while RF performed best with acetonitrile-diluted data, demonstrating how different algorithms complement one another in optimizing detection.

In addition, Yan et al. [181] designed a SERS-based lateral flow assay (SERS-LFA) for the sensitive detection of *E. coli* O157:H7. The system employed antibody-conjugated gold–silver core–shell nanotags loaded with DTNB reporter molecules, which produced strong Raman signals at the test line. To overcome the problem of conventional linear regression in handling complex spectral variations, they integrated sophisticated ML models, including support vector regression (SVR) and extreme gradient boosting regression (XGBR) for quantitative analysis. Impressively, the XGBR model recorded a detection limit of 6.94 × 10^1^ CFU/mL, nearly four orders of magnitude lower than the visual readout threshold, and successfully quantified *E. coli* O157:H7 spiked into milk and beef samples at ultra-low levels after only 2 h of incubation.

## 6. Current Challenges and Future Directions in AI-Assisted Biosensing

Despite remarkable progress, the widespread deployment of AI-assisted biosensors for foodborne pathogen detection is facing critical barriers. Overcoming these problems is important for translating laboratory prototypes into scalable, reliable tools for food safety monitoring. The convergence of AI with biosensor technologies, supported by advances in microfluidics, IoT, and cloud computing, provides unprecedented opportunities to reshape how food systems are monitored. Dealing with current limitations while leveraging these technological synergies will be critical in charting the future directions of AI-enabled biosensing.

### 6.1. Data Standardization and Interoperability

The absence of standardized datasets and reporting protocols is a major barrier to advancing AI-enabled biosensing for foodborne pathogen detection. Qian et al. [201] highlighted this limitation through the Cornell Food Safety ML Repository, underscoring how the lack of well-curated, publicly available datasets continues to hinder AI/ML model development. Similarly, Khoiri and Moussango [202] emphasized the need for harmonized protocols, predictive modeling, and interdisciplinary collaboration. Kotsiri et al. [203] further observed that most biosensors still exhibit limited sensitivity and specificity in complex food matrices, which makes the creation of standardized datasets even more critical. The diverse physicochemical properties of pathogens, combined with interfering substances, complicate biosensor signal interpretation and limit reproducibility across platforms [11,193].

Multi-mode biosensors are emerging as one potential solution by integrating complementary techniques such as Raman spectroscopy, fluorescence, colorimetric, and electrochemical sensing to generate richer datasets that capture multiple pathogen signatures [204,205]. Yet, interpreting such heterogeneous outputs is difficult without computational support. AI algorithms, particularly those employing attention mechanisms, can help by prioritizing strong, reliable signals while attenuating the influence of noisy or weak data [206].

Recent frameworks for AI-assisted monitoring propose standardized database architectures that can be adapted to food safety applications. A robust design should include pipelines for data ingestion, validation, curation, and storage of raw and processed biosensor outputs in unified formats. Metadata standards are also necessary to capture contextual information such as biosensor type, food matrix, and experimental conditions to ensure comparability across studies. Such systems should incorporate algorithm repositories and benchmarking tools, enabling researchers to evaluate and compare AI models on shared datasets [207,208]. Cloud-based, open-access repositories aligned with FAIR principles (findable, accessible, interoperable, and reusable) would further guarantee scalability and interoperability across heterogeneous biosensor platforms [209,210].

Building and sustaining such infrastructure will require international collaboration. A consortium that unites academic institutions, food safety agencies, industry stakeholders, and intergovernmental organizations such as FAO, WHO, or Codex Alimentarius could provide governance, data-sharing standards, and long-term sustainability. Lessons from successful global repositories such as GenBank show how shared governance ensures transparency, trust, and continuous updating [211]. Applying this approach to food safety would reduce geographic and matrix bias while accelerating the development of AI-enabled multimodal biosensors through richer, more representative datasets.

Future research should therefore prioritize the creation of large-scale, curated, open-access biosensor databases supported by harmonized validation protocols and international governance frameworks. Such initiatives will enhance reproducibility, strengthen interoperability, and reduce duplication of effort, thereby laying the groundwork for globally trusted AI-enabled biosensing in food safety monitoring.

### 6.2. Algorithmic Bias and Generalizability Across Food Types

The effectiveness of AI models in food safety applications is fundamentally constrained by the representativeness of training datasets. When datasets are biased toward specific food types or contamination scenarios, models usually underperform when applied to new food matrices or pathogen targets [201,212]. Limited availability of well-curated, publicly accessible datasets underpins this problem, as unbalanced data distributions, such as abundant host records but few infected samples, lower model accuracy, and generalization [213]. Standardized data analysis frameworks are therefore important to mitigating these biases and ensuring reliable performance across contexts [214].

The diverse physical and chemical properties of foodborne pathogens, combined with interfering substances such as biofilms in food matrices, complicate biosensor detection [215]. Sensitivity and specificity typically vary depending on the matrix type, pathogen species, and biosensor surface materials. Although nanoparticle-based systems and advanced biorecognition elements have improved detection performance, the need for universal biosensors capable of functioning reliably across heterogeneous food matrices without requiring extensive matrix-specific adaptations is warranted [22,82].

Recent advances showcase that such platforms are achievable. Wang et al. [30] applied ML models with dual ANN to a non-specific optical sensor array, which enables the identification of multiple pathogenic bacteria in contaminated milk samples despite matrix complexity. Similarly, Quan et al. [35] designed a DL-enhanced digital microfluidic platform for multiplex determination of viable foodborne pathogens, combining robustness against matrix interference with broad-spectrum pathogen detection. These examples demonstrate how AI-driven biosensing is advancing toward universal detection platforms that can overcome matrix variability and pathogen diversity. Moving forward, aligning such innovations with the broader future direction of developing standardized, generalizable, and matrix-independent AI-biosensors is essential to strengthening the practical applicability and reliability of these technologies across diverse food systems.

### 6.3. Cost, Scalability, and Commercialization Barriers

Despite notable advances in consumer electronics, microfluidics, and nanotechnology that have enabled increasingly portable and cost-efficient devices, most AI-enabled biosensors are confined to research laboratories. High fabrication costs, limited scalability, and reliance on skilled personnel continue to obstruct translation into real-world food safety monitoring. The use of advanced bioreceptors such as aptamers and engineered enzymes, coupled with high-precision transducers, elevates production costs, while AI integration imposes additional computational demands. These challenges are more pronounced in low-resource regions, where food safety risks are greatest but access to high-end infrastructure is limited. Moreover, many AI applications demand substantial processing power, large datasets, and reliable connectivity, further constraining adoption outside controlled laboratory settings and limiting practicality for non-professional users or decentralized testing environments [119,216].

To address these barriers, research is increasingly focused on low-cost, scalable AI-biosensors through both hardware and computational innovation. Paper-based colorimetric platforms integrated with smartphone AI applications provide affordable, point-of-care detection for resource-limited contexts [217]. Triboelectric nanogenerators coupled with AI (AI-TENGs) harvest energy from motion or environmental stimuli, creating self-powered systems that reduce dependence on external power supplies and enhance portability [218]. Aptamer-based sensors, optimized by AI for high biomarker specificity, are being miniaturized through integration with micro-ICs, enabling compact devices for real-time diagnostics and continuous monitoring [219]. Bio-based substrates such as cellulose, chitosan, and silk fibroin are also gaining attention as sustainable, biodegradable materials that reduce fabrication costs while broadening accessibility [220]. Complementary advances include IoT-based architectures that centralize computational tasks, cutting system costs by an estimated 25% [221], computer-aided design (CAD)-enabled pipelines that simplify prototyping and accelerate cell-free biosensor development [222], and edge-computing chips that allow localized AI analysis, reducing reliance on costly cloud infrastructure while enabling real-time, on-site decision-making [223].

Future progress will depend on advances in material science, scalable manufacturing, and edge-intelligent diagnostics to ensure robust yet affordable performance. Lowering production costs, improving portability, and simplifying operation are critical to expanding adoption beyond laboratory prototypes and into diverse settings. A coordinated effort that integrates sustainable materials, mass-production strategies, and localized data processing has the potential to transform AI-enabled biosensors from experimental tools into practical, accessible, and reliable systems for pathogen detection.

### 6.4. Regulatory Acceptance and Validation Requirements

Regulatory acceptance is one of the most formidable hurdles for AI-enabled biosensors in food safety monitoring. Unlike conventional culture-based assays with established benchmarks, AI-driven platforms introduce algorithmic decision-making that is often opaque, giving rise to the so-called “black-box” problem. This lack of transparency raises concerns about reproducibility, accountability, and liability in cases of false negatives or positives, which makes regulators cautious about approving these systems for routine use where public health is directly impacted [212,224].

While ML-enhanced biosensors have shown considerable promise in detecting a wide range of contaminants [170,225], their interpretability is still restricted. The inability to explain how models arrive at predictions undermines trust and complicates compliance [212,224]. Explainable AI (XAI) frameworks are beginning to address this gap. For example, Local Interpretable Model-Agnostic Explanations (LIME) perturbs input data to build surrogate models, providing rapid, instance-level insights that can vary across runs. By contrast, SHapley Additive Explanations (SHAP) applies cooperative game theory to assign contribution scores to individual features, delivering both local and global interpretability at the cost of greater computational demand. The What-If Tool (WIT) adds value by enabling interactive, code-free inspection of model behavior. Comparative analyses indicate that while LIME and WIT offer rapid, user-friendly insights, SHAP provides more computationally intensive but robust and comprehensive explanations, making these tools complementary in enhancing the transparency of food fraud detection models [226]. However, despite these advances, XAI remains in its infancy in food safety applications and has yet to deliver fully regulatory-compliant solutions.

Another regulatory complication arises from the dynamic nature of AI models, which evolve via retraining and continual optimization. Traditional approval pathways are not designed to accommodate such fluid systems [119]. Despite this, precedents from other diagnostic domains demonstrate that regulatory adaptation is feasible. Notably, Roche’s Accu-Chek SmartGuide, an AI-enabled continuous glucose monitoring biosensor, has received CE marking in the European Union, confirming conformity of both its biochemical sensing hardware and predictive AI features with regulatory standards [227]. This case highlights that algorithm-enhanced biosensors can achieve formal approval when accompanied by robust validation protocols. In contrast, AI-based biosensors for foodborne pathogens remain at the proof-of-concept stage, with none yet advancing to market approval.

Future directions must therefore prioritize the creation of adaptive regulatory frameworks that explicitly incorporate AI integration. This includes developing explainable and auditable AI methods to strengthen transparency, establishing harmonized validation protocols with algorithmic performance metrics, and fostering early collaboration between researchers, regulators, and industry stakeholders to design certification pathways that balance innovation with accountability. Such efforts align with the vision of Food Safety 4.0, in which intelligent, connected systems enable real-time monitoring and global trust [21,228,229].

### 6.5. Data Privacy and Security Concerns

The integration of biosensors with cloud platforms and IoT infrastructures allows for real-time tracking and big data analytics, but exposes sensitive information to cybersecurity risks [74]. Food supply chain data, covering contamination events, production sites, and consumer markets, is commercially and strategically valuable, which makes it a target for malicious attacks [230]. In addition, compliance with data privacy laws such as the General Data Protection Regulation (GDPR) is essential when health-related information is involved [231]. Future efforts should prioritize secure data transmission via end-to-end encryption, blockchain-based traceability for tamper-proof records, as well as decentralized architectures that minimize vulnerabilities. Embedding cybersecurity considerations during the design stage, rather than as afterthoughts, will be critical for industry confidence and public trust.

## 7. Conclusions

Ensuring food safety in an increasingly globalized supply chain necessitates rapid, accurate, and scalable pathogen detection strategies. Traditional culture-based methods, while reliable, are too slow to meet modern demands, whereas advanced molecular and immunological approaches are constrained by several issues of sensitivity, reproducibility, and field applicability. Biosensors have emerged as promising tools to overcome these barriers by providing portability, rapid response, and integration into real-time monitoring systems. Nevertheless, problems such as matrix interference, limited sensitivity across diverse food types, high fabrication costs, as well as regulatory hurdles continue to constrain their widespread adoption.

Artificial intelligence is a powerful avenue to resolve many of these barriers, as it enables advanced signal processing, improves sensitivity and specificity, and allows reliable pathogen classification across heterogeneous food matrices. In particular, machine learning models such as support vector machines and convolutional neural networks have been successfully applied to analyze complex biosensor outputs, thereby allowing for swift and highly accurate identification of pathogens in food samples. AI-driven multimodal data fusion, as well as incorporation with IoT and edge computing, are redefining the potential of biosensors from proof-of-concept prototypes to scalable diagnostic solutions. Early applications, including AI-enhanced optical arrays and digital microfluidic platforms, demonstrate how these innovations can simultaneously manage pathogen diversity and matrix complexity, thus moving the field closer to universal detection platforms.

The successful translation of AI-assisted biosensors from laboratory to field, however, depends on coordinated efforts in data standardization, cost-efficient manufacturing, regulatory alignment, and cybersecurity protection. Interdisciplinary collaboration from food microbiology, material science, computer science, and regulatory policy will be vital for overcoming current challenges and accelerating practical deployment. The integration of biosensor technologies with the analytical power of AI makes the vision of Food Safety 4.0 for interconnected, intelligent, and predictive food monitoring systems increasingly attainable. It holds the potential to transform global food safety assurance by delivering rapid, reliable, and scalable pathogen detection across diverse food samples to protect both consumers and public health.

## Figures and Tables

**Table 1:** Comparison of various pathogen detection technologies.

Method	Detection Time	Sensitivity	Cost and Resources	Portability	Regulatory Acceptance	Advantages	Limitations	References
Culture-based methods	Days	Very high	Low to moderate; requires media, incubator, Biosafety Level 2 (BSL-2), and trained personnel	Lab-based	Gold standard; widely accepted (reference methods in BAM/ISO)	Species/viability confirmation; inexpensive consumables	Time-consuming; cannot detect VBNC pathogens	[55,57]
Biochemical test	Days	Moderate	Low; requires culture step and reagents	Lab-based	Widely used for presumptive identification	Simple, low cost	Requires pure isolates; limited specificity; only provides presumptive identification	[42,65]
ELISA	Hours	Moderate	Low to moderate; plate reader or lateral-flow kits	Lab-based; Benchtop; LFA portable	Accepted for screening; confirmation needed	Scalable, user-friendly	Susceptible to cross-reactivity and food-matrix interference; often requires enrichment; limited to known antigens	[50,66]
PCR/qPCR	Hours	High	Moderate per test; high capital cost for thermocycler and other equipment	Lab-based but portable versions exist; mainly benchtop	Broad acceptance (ISO)	High sensitivity/specificity; multiplexing	Inhibited by complex food matrices, detects DNA from dead cells, requires skilled personnel, and a controlled environment	[55,67]
Next-Generation Sequencing	Days	Very High	High sequencing platforms and bioinformatics	Lab-based	Emerging outbreak/source-tracking	Comprehensive profiling, strain-level identification	High cost and turnaround; requires advanced bioinformatics; not feasible for routine quality control	[68,69]
Conventional biosensor	Minutes to Hours	Moderate to High	Low to moderate; portable reader	Portable handheld devices, suitable for field use	Limited; assay-specific validation	Rapid, simple, portable	Signal drift and noise in complex samples; calibration and standardization issues; limited regulatory validation	[70,71]
Microfluidics/Lab-on-a-chip	Minutes to Hours	High	Moderate; chip and reader, fabrication required	Compact, field-deployable chip-based modules	Limited; emerging	Low reagent use; integrates preparation and detection	Device fabrication and reproducibility challenges; prone to clogging/fouling; requires specialized fabrication facilities	[72,73]
IoT-enabled devices	Real-time/continuous	Variable	Moderate; sensors and connectivity	Remote, real-time monitoring across the supply chain	Limited; complementary only	Remote monitoring; traceability	Indirect detection of hazards; data security and connectivity issues; confirmatory tests still required	[74,75]
CRISPR-Cas-based molecular diagnostics	Minutes to Hours	Very High	Low to moderate; isothermal setup and Cas reagents	Field-deployable, portable isothermal platforms	Emerging	Ultra-sensitive, specific, rapid	Limited standardization; off-target activity risk; regulatory pathways still emerging	[76]
AI-assisted biosensor	Minutes to Hours	High	High upfront development and hardware costs; needs for computational power, specialized software, skilled personnel, and extensive training datasets	Portable sensor platforms	Emerging	Improved detection in noisy matrices; automated decision support	Model interpretability (“black box”) issues require large datasets and revalidation; regulatory acceptance is still limited	[77,78]

**Table 2:** Applications of AI-integrated biosensors for foodborne pathogen detection across different food matrices.

Food Matrix	Biosensor Type	AI Method	Detection Principle	Target Pathogen	Performance Metrics	References
Milk	Nanogap-assisted hybrid biosensor	Machine learning (ML)—bootstrapping soft shrinkage–partial least squares regression	PCR amplification of *nuc* gene captured in Au/Ag nanogaps; nanogap “hotspots” enhance SERS signals; ML model improves spectral analysis and quantitative prediction	*Staphylococcus aureus*	Root mean-square error of prediction: 0.437; prediction set correlation coefficient: 0.967	[171]
Orange/strawberry juice, milk	Photoelectrochemical	ML (with molecular docking)	BIPs constructed with *S. aureus* and 4-ethynylacetophenone; dual-mode operation: active bias enhances electron transfer, passive bias repels cells; ML used to analyze and predict sensor performance, reducing background interference	*S. aureus*	LOD: 10^1^ CFU/mL; High specificity vs. other bacteria	[146]
Milk, chicken	Fluorescence	Convolutional neural networks (CNNs)	QD–aptamer probe fluorescence imaged inside microchannel; CNN processes images to quantify *E. coli* concentration and filter noise	*Escherichia coli*	LOD: 2 CFU/mL; Linear range 10–3 × 10^6^ CFU/mL (R^2^ = 0.990); 100% capture at 4 × 10^2^ CFU/mL; >99% accuracy; Recovery 96.7–104%	[155]
Tap water	Fluorescence	Support vector machine	PQD-based array detects fluorescence quenching from pathogen interactions; ML models classify and quantify species and mixtures; simultaneous detection and inactivation	*E. coli*, *S. aureus*, *Salmonella typhimurium*, *Listeria monocytogenes*, *Pseudomonas aeruginosa*	LOD: 93–136 CFU/mL; accuracy: 100% for individual/mixed pathogens; antibacterial efficacy: >99% in 30 min	[156]
Water, coconut juice	Fe–N–C single-atom nanozyme (SAzyme)-based colorimetric sensor array	Machine learning (Principal Component Analysis, Linear Discriminant Analysis, and Hierarchical Clustering Analysis)	Fe-N-C single-atom nanozymes catalyze chromogenic substrates, producing color changes. Pathogens inhibit the SAzymes’ activity, resulting in distinct colorimetric signals. ML processes these signals to create unique fingerprints for each pathogen, enabling differentiation and identification	*S. aureus*, *S. enterica*, *Vibrio vulnificus*, *V. harveyi*, *L. monocytogenes*, *V. parahaemolyticus*	Detection range: 10^5^–10^8^ CFU/mL; simultaneous detection; stable over a period of 25 days	[172]
Tap water, apple juice	LC-based aptasensor	Artificial Neural Network (ANN) (water), XGBoost (juice)	Liquid crystal alignment change captured by polarized microscopy; ML models analyze image features for sensitive classification and quantification	*E. coli*	LOD: 6 CFU/mL; R^2^: 0.986 (water), 0.976 (juice); Detection ~5 min	[157]
Tap water, pork	Fluorescence	K-nearest neighbors (KNN), naive Bayes (best), decision tree, linear discriminant analysis (LDA), support vector machine (SVM)	Fluorescence quenching from CQD–bacteria binding generates unique signal patterns; ML classifies fingerprints for species and mixture ID	*E. coli* O157:H7, *S. aureus*, *P. aeruginosa*, *Shigella*, *L. monocytogenes*	LOD: 10^3^ CFU/mL; detection in 5 min; 100% accuracy in real and mixed samples; high anti-interference	[131]
Fresh produce	Optical	ML (random forest and support vector machine)	Photonics-based sensor system generating optical signals for pathogen detection, with ML algorithms analyzing the signals to predict contamination risk	*E. coli*, *Salmonella enterica*	Accuracy up to 95%; F1-score > 0.9; high sensitivity	[77]
Milk	ssDNA–nanoparticle optical sensor array	Partial least square discriminant analysis (PLS-DA), KNN, RF classifier, SV classifier, multilayer perceptron (MLP), Kolmogorov–Arnold network (KAN)	Pathogen biomolecules displace ssDNA from nanoparticles, restoring fluorescence; ML models classify fluorescence fingerprints for species ID	*E. coli*, *S. enterica*, *S. aureus*, *Shigella sonnei*, *Bacillus cereus*	Accuracy up to 98.4% (MLP, 120 min); >90% at 30 min; high anti-interference	[30]
Shredded cheddar cheese	Colorimetric	Artificial neural network (deep feed-forward neural network)	PCA detects pathogen-specific volatile organic compounds (VOCs) via color changes; ANN decodes complex VOC-induced patterns to distinguish pathogens from high background flora	*S. enteritidis*, *E. coli* O157:H7	Accuracy: 85–92% (3–5 log CFU/g), 72–96% at 1 log CFU/g within 1 day; no enrichment or incubation required	[173]
Ground chicken	Colorimetric	Deep feed-forward neural network	Pathogen-specific VOC emissions interact with chromogenic dyes; ML recognizes temporal colorimetric shifts for species identification and multiplex detection	*L. monocytogenes*, *S. enterica*, and *E. coli* O157:H7	Accuracy: >90% at levels as low as 1 log CFU/g within 5–7 h (25 °C); >80% accuracy within 24 h at 4 °C	[174]
Orange juice, fish, milk, drumsticks, lettuce, chicken, streaky pork	Fluorescence	Elastic network regression	Phages capture viable cells, DNA cleaved by CbAgo, fluorescent probes targeted, signals accumulated on microsphere; machine vision interprets signals for accurate quantification	*S. typhimurium*	LOD: 40.5 CFU/mL; Recovery: 93.22–106.02%; Coefficient of variation: 1.47–12.75%	[38]
Blueberries	Colorimetric	Deep learning (DL)—YOLOv5	HAase secreted by live bacteria degrades CPRG-loaded HA hydrogel; released CPRG reacts with β-gal hydrogel, and color change is analyzed via YOLOv5	*E. coli*, *P. aeruginosa*, *S. aureus*, Group A *Streptococcus*	LOD: 10 CFU/mL; detection time: 60 min; accuracy: >92%	[175]
Meat products	Electrochemical	Cloud-based algorithm (feature extraction + statistical classification)	Membrane-engineered cells detect shifts in potential upon pathogen binding; the algorithm processes signals for classification	*Salmonella* spp.	Accuracy: 86.1%; LOD: 1 log CFU g^−1^; Time-to-result; could detect the pathogen within 24 h after a 3 min analysis	[176]
Milk	Nanosensor array (aggregation-induced emissive nanosilicons)	XGBoost, ANN	Nanosilicon array differentiates pathogens based on surface potential and hydrophobic interactions; AI enables classification and quantification of multiple pathogens simultaneously	*E. coli*, *Cronobacter sakazakii*, *L. monocytogenes*, *S. enteritidis*, *V. parahaemolyticus*, *S. aureus*, *Campylobacter jejuni*, and *Shigella dysenteriae*	Accuracy: 93.75–100% within 1 h; Quantification limits: 10^3^ CFU/mL (*C. sakazakii*), 10^2^ CFU/mL (*L. monocytogenes*); Mixed-sample detection: 10^5^ CFU/mL	[148]
Milk and seawater	Electrochemical	Random forest	Whole-cell imprinted polymer on electrode records impedance signals; six EIS parameters processed by ML for classification and semi-quantification	*E. coli*, *S. aureus*, *V. parahaemolyticus*	Detection range: 10–10^6^ CFU/mL; accuracy: 95.00%	[177]
Peanuts, maize	Whole-cell biosensor	Random forest (best), sPLS-DA, SVM, ANN, HDDA	VOCs from mold infection trigger promoter responses; biosensor luminescence patterns classified using ML to detect mold presence and stage	*Aspergillus flavus*	Accuracy: 100% for healthy vs. infected, 95% for pre-mold stages in peanuts, 98% for pre-mold stages in maize, 83% for infected peanuts vs. maize; detection Time: Up to 6 h	[167]
Milk	Electrochemical	Decision tree models	Differential impedance responses from multilayer films analyzed with ML; multidimensional calibration spaces discriminate pathogen concentrations, interferences, and mastitis infection states	*S. aureus*	LOD: 2.01–3.41 CFU/mL; Accuracy: 100% (spiked blank milk), 94% (crude milk, multiclass), 100% (ternary classification of infected, treated, and healthy samples)	[178]
Spoiled food simulations	Raman spectroscopy-based fiber-optic sensor	ANN, RF, SVM, KNN, XGBoost, LR	The fiber-optic Raman probe excites pathogen-specific VOCs with a 532 nm laser, and the scattered light is captured by a spectrometer. ML analyzes the raw Raman spectra to extract molecular features and improve detection accuracy	*L. monocytogenes*, *S. typhimurium*, and *E. coli*	ANN: 95% accuracy (AUC = 0.99); RF: 85% accuracy at 100-fold dilution	[179]
Fresh-cut romaine lettuce	Colorimetric	Machine learning-enabled multilayer neural network	VOC emissions from viable pathogens generate dye-specific colorimetric patterns; ML recognizes patterns for species-level identification, viability discrimination, and multiplex detection	*E. coli* O157:H7, *S. aureus*, *L. monocytogenes*	Accuracy: 95% for bacterial identity, 93% for quantification, 91% for multiplexed samples; Detection time: Visible pattern change within 2 h	[78]
Milk	Fluorescence	DL—Faster Region-Based Convolutional Neural Network	Immunomagnetic capture and fluorescent labeling; low-gradient magnetic field converts signals to planar distribution; DL algorithm identifies fluorescent spots for accurate quantification	*S. typhimurium*	Limit of detection: 55 CFU/mL; Linear range: 69–1100 CFU/mL; Recovery: 85.31–110.48% (mean 102.74%); Coefficient of variation: < 8%; Detection time: 2.5h	[180]
Milk and Beef	SERS-LFA (surface-enhanced raman scattering-based lateral flow assay)	Bayesian ridge regression (BRR), elastic net regression (ENR), support vector regression (SVR), and eXtreme gradient boosting regression (XGBR)	Double-antibody sandwich immunoassay with SERS nanotags; ML regression models used for quantitative analysis of Raman spectra	*E. coli* O157:H7	LOD: 6.94 × 10^1^ CFU/mL; Recovery: 86–128%; Detection possible after 2 h incubation	[181]

## Data Availability

No data associated with this study.

## Abbreviations

The following abbreviations are used in this manuscript:

WHO	World Health Organization
AI	Artificial Intelligence
CNNs	Convolutional Neural Networks
SERS	Surface-Enhanced Raman Spectroscopy
IoT	Internet of Things
NGS	Next-Generation Sequencing
VBNC	Viable but Non-Culturable
LFDs	Lateral Flow Devices
PCR	Polymerase Chain Reaction
IUPAC	International Union of Pure and Applied Chemistry
MIPs	Molecularly Imprinted Polymers
SELEX	Systematic Evolution of Ligands by Exponential Enrichment
Nb-HRP	Nanobody–Horseradish Peroxidase
EIS	Electrochemical Impedance Spectroscopy
SPR	Surface Plasmon Resonance
QCM	Quartz Crystal Microbalance
ML	Machine Learning
DL	Deep Learning
RF	Reinforcement Learning
ANN	Artificial Neural Network
LPS	Lipopolysaccharide
CQDs	Carbon Quantum Dots
k-NN	k-Nearest Neighbors
BIPs-PEC	Bacterial Imprinting Photoelectrochemical
RNNs	Recurrent Neural Networks
SVM	Support Vector Machine
VOCs	Volatile Organic Compounds
SHDS	Staggered Herringbone Double-Spiral
PQD	Perovskite Quantum Dot
LFA	Lateral Flow Assay
SVR	Support Vector Regression
XGBR	Extreme Gradient Boosting Regression
CAD	Computer-Aided Design
XAI	Explainable Artificial Intelligence
